# Draft Genome Sequence of *Rhodococcus enclensis* 23b-28, a Model Strain Isolated from Cloud Water

**DOI:** 10.1128/genomeA.01199-17

**Published:** 2017-10-26

**Authors:** A. Lallement, L. Besaury, B. Eyheraguibel, P. Amato, M. Sancelme, G. Mailhot, A. M. Delort

**Affiliations:** Université Clermont Auvergne, CNRS, Institut de Chimie de Clermont-Ferrand, Clermont-Ferrand, France

## Abstract

The whole genome of *Rhodococcus enclensis* 23b-28, a bacterial strain isolated from cloud water, was sequenced. This microorganism is equipped with genes able to degrade aromatic compounds and could thus play a role in complex organic matter decomposition in cloud water.

## GENOME ANNOUNCEMENT

Phenol can be found in all environmental compartments (soil and water), including the atmosphere (1, 2). Due to its high volatility, phenol is present in the gas phase, but this compound can also be transferred to the aqueous phase of the atmosphere (rain, snow, and clouds) thanks to its solubility (defined by Henry’s law constant). The degradation of organic matter in cloud water occurs through photochemical reactions due to solar light, but also through enzymatic reactions performed by viable microorganisms (3) that can survive in clouds despite the numerous stresses they encounter (oxidative, radiative, and osmotic stresses) (4). Among the different strains isolated from cloud water from the Puy de Dôme station (1,465 m above sea level, France) the strain *Rhodococcus enclensis* 23b-28 was one of the most efficient for phenol degradation. This strain has been isolated aerobically, has the ability to develop at low temperature (17°C), and can produce biosurfactants (5). To further understand the specificities of this strain, its ability to degrade aromatic compounds, its presence, and the implications for cloud biochemistry, its whole genome was sequenced. Whole-genome shotgun sequencing (2 × 150 bp) was prepared using the Nextera DNA sample preparation kit (Illumina, San Diego, CA, USA), following the manufacturer’s user guide, and sequencing was done on an Illumina MiSeq sequencer (MR DNA, Shallowater, TX, USA). Sequence data files were filtered for quality using FastQC (https://www.bioinformatics.babraham.ac.uk/projects/fastqc/), trimmed using Prinseq-Lite (6), and *de novo* assembled with SPAdes (7). A total of 118 contigs were generated, with an average coverage of 23.1-fold. The average contig size was 61,133 bp, and the *N*_50_ contig size was 158,305 bp. The size of the assembled genome is 7,213,744 bp, with a GC content of 62.3% (this is a low value for *R. enclensis*, for which the GC content is 66.9% [8], but it is in the range of those obtained for *Rhodococcus* species in general, at 62 to 69% [9]). The RAST annotation server was used for annotating the draft genome of *Rhodococcus enclensis* 23b-28 (http://rast.nmpdr.org). It contains 59 RNAs and 6,755,000 protein-coding genes, of which 34% were assigned to a total of 2,367 subsystems; among those, 96 were affiliated with aromatic degradation. Genetic sequences affiliated with enzymes involved in benzoate degradation were found, along with the two degradation pathways for catechol (ortho- and meta-). This information allows us to think that *Rhodococcus enclensis* 23b-28 can play a role in the degradation of aromatic compounds in clouds.

### Accession number(s).

This whole-genome shotgun project has been deposited in the GenBank database under the accession number NOVD00000000.
